# Autoimmune syndrome induced by adjuvants after breast enhancement with polyacrylamide hydrogel: a study in Poland

**DOI:** 10.1007/s00296-020-04605-5

**Published:** 2020-05-24

**Authors:** Ewa Woźniak-Roszkowska, Maria Maślińska, Piotr Gierej, Bartłomiej Noszczyk

**Affiliations:** 1 Department of Plastic Surgery, Prof. W. Orłowski Memorial Hospital, Centre of Postgraduate Medical Education, Czerniakowska 231, 00-416 Warsaw, Poland; 2 National Institute of Geriatrics, Rheumatology and Rehabilitation, Early Arthritis Clinic, Spartańska 1, 02-637 Warsaw, Poland

**Keywords:** Hydrogel, Adjuvants, Breast implants, Autoimmunity

## Abstract

Autoimmune syndrome induced by adjuvants (ASIA) is the spectrum of diseases in which the substances considered inert to the body induce autoimmune reactions and inflammation. Some of the biomaterials recently used in plastic surgery, such as silicone or polyacrylamide hydrogel (PAAG) seem to trigger clinical features of ASIA. The aim of this study was to assess the incidence of these features within a group of women after breast augmentation with PAAG. As many as 30 consecutive patients (26–59 years, mean age 39.5) referred to the Clinic of Plastic Surgery after breast enhancement with PAAG were examined. The validated criteria of ASIA syndrome were employed. Descriptive statistics were chosen based on the distribution of variables. The research was approved by the Bioethical Committee of the Centre of Postgraduate Medical Education in Warsaw, Poland. Within the studied group, 50% of patients (*n* = 15) fulfilled ASIA diagnostic criteria. Apart from local complications, we encountered various general symptoms, among which fever (*n* = 13, 43.3%), tingling and numbness of upper extremities (*n* = 10, 33.3%) and chronic fatigue (*n* = 9, 30%) were the most common. These symptoms were present on an ambulatory visit, before qualification to the operation of hydrogel removal. All patients undergoing surgical PAAG removal (*n* = 8) declared alleviation or complete resolution of the symptoms. Polyacrylamide hydrogel breast filling, although limiting the invasiveness of the procedure in relation to silicone breast implants, also carries the risk of developing ASIA symptoms. The removal of PAAG may bring improvement in some cases.

## Introduction

Autoimmune/autoinflammatory syndrome induced by adjuvants (ASIA) is a spectrum of immune-mediated symptoms and conditions that occur as a result of chronic stimulation of the immune system by the adjuvant agent [1, 2]. Adjuvant is a non-specific stimulating substance able to increase either the cellular or humoral response to the presence of an antigen [1]. The possible adjuvants can be classified into oil emulsifications, such as Freund’s adjuvant, paraffin oil or processed petroleum jelly, minerals (silicone dioxide, beryllium, aluminium, calcium compounds), or bacterially derived particles (Staphylococcus, Nocardia, Salmonella, Mycobacterium) [3]. The first descriptions of autoimmune phenomena after adjuvants come from the 1950s and 1960s, from animal models [4, 5] for follow-up in augmentation mammoplasty [3, 6–13]. In 2011 Shoenfeld et al. [1] proposed a classification for diagnosis of ASIA which requires the fulfillment of at least one major or one major and two minor criteria. Major criteria of ASIA syndrome include exposure to external stimuli (infection, vaccine, silicone, adjuvants) before the emergence of clinical symptoms; presentation of at least one of the following clinical symptoms: muscular weakness, myalgia or myositis, arthritis or arthralgia, chronic fatigue, unrefreshing sleep or sleep disorders, neurological manifestations, fever and dry mouth; improvement after removal of the adjuvant and a typical result of the biopsy of affected organs revealing local cutaneous inflammation with silicone granuloma formation and high level of IgG in the surrounding tissue [2]. As minor ASIA criteria the authors considered the presence of autoantibodies or antibodies directed at the suspected adjuvant, other clinical manifestations (e.g. irritable bowel syndrome) and the presence of specific HLA (i.e. HLA DRB1, HLA DQB1) [1, 2].

Siliconosis after classical breast implants is one of the most common types of ASIA [6–14].

Polyacrylamide hydrogel (PAAG), obtained by polymerization of acrylamide monomer into viscoelastic gel, is a material that has been increasingly used in plastic surgery. The gel composed of 2.5–5% polyacrylamide water solution, with pH 7.5, was first used as a tissue filler by Ukrainian surgeons in 1980 for breast augmentation. Other PAAG treated areas included lips, nasolabial fold and malar area [15, 16]. After it was approved for use in China as a permanent filler for breast augmentation, approximately 200,000 women underwent the procedure between 1997 and 2006 [17]. The most common reported PAAG side effects were those of local character (breast pain and oedema, palpable masses, hematoma, asymmetry, gel migration, and infection) [15, 18]. The increasing incidence of complications after this procedure as well as potential neurotoxic and carcinogenic effect of polyacrylamide raise a concern about the possibility of general toxic symptoms and autoimmune reactions as well [19].

The use of polyacrylamide hydrogel in Poland began in 2012. The exact incidence of use of intra-breast injections in general population is unknown, due to the lack of statistics (the procedure was performed only in private practices). It can be estimated that up to a few thousands of patients could undergo this procedure until 2019 when the distributor was forced to cease the trade due to the growing number of reported complications [20]. The Department of Plastic Surgery at the Centre of Postgraduate Medical Education in Warsaw is a tertiary level center and so far has treated the largest number—among all public departments in Poland—of women suffering from complications after breast enhancement with polyacrylamide.

## The aim of the research

The aim of the study was to assess the incidence of clinical features of ASIA within a group of women after primary minimally invasive procedure (breast augmentation with polyacrylamide hydrogel—PAAG) performed in other medical centers (outpatient clinics and private practices) and subsequently admitted to the Department of Plastic Surgery, Prof. W. Orłowski Memorial Hospital, at the Centre of Postgraduate Medical Education in Warsaw in the years 2016–2019, with complaints and symptoms suggestive for ASIA syndrome.

## Methodology

The analyzed group included 30 women who were referred to the Department of Plastic Surgery for one of the following reasons: the need for immediate surgical treatment of acute complications; chronic symptoms after hydrogel injection; asymptomatic women anxious about possible complications in the future. Only the patients after breast augmentation were taken into consideration, which enabled the objective comparison of the presented symptoms.

The exclusion criteria were as follows:

- the injection of polyacrylamide hydrogel into body areas other than breast (i.e. buttocks or face),
- breast augmentation with PAAG followed by silicone implants,
- the presence of autoimmune disorders in the medical history of study participants prior to PAAG breast enhancement.

Demographic data as well as the information concerning implantation procedures (setting, number of injections, volume of injected hydrogel), were obtained in each case. Table 1 shows the characteristics of the study group.

**Table 1:** Characteristics of the study group

Variable	Value
Age (years), mean ± SD	39.54 ± 8.50
Time from implantation to onset of complications (months), median (IQR)	12 (34.5)
Volume of injected hydrogel (ml), median (IQR)	300 (190)
Number of injections, median (IQR)	1 (0.75)

*IQR* interquartile range, *SD* standard deviation

Based on the past medical history and current physical examination, both local and systemic symptoms before and after the procedure were evaluated. Patients from the studied group underwent laboratory tests depending on the clinical situation (complete blood count, erythrocyte sedimentation rate, C-reactive protein level, antinuclear antibodies, rheumatoid factor, thyroid profile). In cases requiring surgical treatment, samples from the involved tissues (e.g. from fat tissue, pectoralis muscle) were collected for histopathological examination. The cases were subsequently verified as to whether they met the proposed ASIA syndrome criteria.

The research was approved by the Bioethical Committee of the Centre of Postgraduate Medical Education in Warsaw, Poland (resolution no. 25/PB/2019 of 10 April 2019). The informed consent was obtained from all patients.

### Statistical analysis

Descriptive statistics were chosen based on the distribution of variables. The Shapiro–Wilk test was used to assess for normality. Frequencies and percentages were used for count data. All analyses were completed in the R software (version 3.5).

## Results

The majority of patients were admitted to the outpatient clinic (*n* = 25) on a scheduled basis, but some of them required immediate treatment of acute complications and were admitted directly to the hospital department (*n* = 5).

Among the whole group (*n* = 30), 2 patients were considered completely asymptomatic at the time of the admission, whereas other (*n* = 28) presented a wide range of either local or general symptoms. The main reasons for seeking the medical attention were local complications, such as mastalgia, breast asymmetry, breast oedema or fistula formation. Patients complained of general symptoms, such as fever, general weakness, sleep disturbances. Median interval between the hydrogel application and the onset of symptoms was 18.2 months (Table 1). In 6 cases, according to medical data revealed by the patients, the symptoms appeared immediately after PAAG injection. These involved mainly local complications, such as breast pain, oedema of subcutaneous tissue, gland and pectoralis muscle (described in ultrasonography or magnetic resonance imaging) with fever and increased inflammation markers (C-reactive protein level up to 200 ng/ml). These manifestations were attributed mainly to acute infection after the PAAG injection procedure. Permanent breast deformities, such as induration or lumps, took longer to appear (up to 43 months, on average 12.7 months). General symptoms occurred usually after a longer interval e.g. chronic fatigue evolved after an average of 27.6 months from the injection (1–60 months); joint pain after 34.5 months (12–57 months), major depressive disorder after an average of 25.8 months (1–60 months). Muscle weakening as well as sleep disturbances appeared after approximately 1 year after the procedure. Recurrent numbness and tingling of upper extremities were very common, concerned one-third of the whole group and occurred on average after 17.8 months. The incidence of local and generalized symptoms is summarized in Tables 2 and 3.

**Table 2:** The incidence of local symptoms

Symptom	Number of patients and percentage
Breast pain	22 (73.3%)
Breast deformities, lumps	19 (63.3%)
Clinically detectable hydrogel migration	16 (53.3%)
Breast infection/abscess	12 (40%)
Axilla pain	11 (36.7%)
Breast oedema	11 (36.7%)
Fistula	11 (36.7%)
Skin discoloration	3 (10%)

**Table 3:** The incidence of general symptoms

Symptom	Number of patients and percentage
Recurrent fever	13 (43.3%)
Upper extremity numbness/tingling	10 (33.3%)
Chronic fatigue	9 (30%)
Major depressive disorder symptoms	9 (30%)
Lymph nodes enlargement	8 (26.7%)
Limitations of upper extremities’ movements	6 (20%)
Bodyweight loss	2 (6.7%)
Artralgia	2 (6.7%)
Increased sweating	1 (3.3%)
Morning joint stiffness	1 (3.3%)
Raynaud’s phenomenon	1 (3.3%)
Increased infection incidence, decreased immunity	1 (3.3%)
Breathing disorders	1 (3.3%)

We compared the aforementioned manifestations with diagnostic criteria of ASIA. As much as 86.7% of all studied patients presented symptoms consistent with major diagnostic ASIA criteria (chronic fatigue, sleep disorders, recurrent fever, arthralgia). The most common symptoms—which are unspecific and also may indicate the involvement of the peripheral nervous system—were hand pain, numbness and tingling as well as excessive sweating (33.3%). These symptoms were not associated with the septic state.

Among patients with symptoms suggesting the development of ASIA syndrome, *n* = 8 (53.3%) underwent surgical treatment with incision of one or both breasts with hydrogel removal, often combined with extensive debridement of necrotic tissues and drainage. Importantly, all patients in this subgroup have experienced a reduction in symptoms severity and five of them confirmed complete symptoms resolution. Histopathological examination of the tissues obtained during the gel removal revealed features of nonspecific inflammation, with the presence of macrophages, muscle and fat tissue necrosis. The histopathological picture did not indicate septic features.

On the basis of the presented symptoms, the diagnosis of ASIA could be confirmed in 50% (*n* = 15) of patients. There were 15 patients who did not fully meet the ASIA criteria, however, 73% of this group (*n* = 11) had one major criterion.

## Discussion

Classically, the spectrum of ASIA encompassed four medical entities: siliconosis, Gulf War syndrome (GWS), macrophage myofascitis syndrome (MMF), and post-vaccination phenomena [2, 21–23]. Their common feature is a cause and effect relationship with the exposure to an adjuvant substance (silicone dioxide, squalene, and aluminum hydroxide, respectively). Another commonly incorporated ailments are chronic fatigue syndrome (CFS), often associated with different sleep disturbances, and sick building syndrome (SBS) [2].

To our knowledge, this is the first study describing a potential ASIA mechanism after polyacrylamide hydrogel intra-breast injections. We were first in Poland to pay attention to the possibility of systemic manifestations even many years after PAAG implantation. Although breast enlargement is a procedure typical for the field of plastic surgery, information about its performance in the past and possible systemic complications may be important for clinicians from various fields of medicine, including rheumatology and immunology.

Vera-Lastra et al. [23] presented research involving the development of ASIA syndrome after illegal injections of different substances for cosmetic purposes. The study involved 50 women who, differently to the present study, were injected with different fillers, mainly with mineral oil (*n* = 41, 82%), but also iodine gadital, guaiacol, collagen or silicone fluid.

In the described work, buttocks were the main site of fillers application (*n* = 36, 72%). The group included only eight women after breast enhancement with illegal fluids. In our study we decided to exclude other than breasts areas of injections, therefore the presented group was homogeneous in terms of injective material used and localization of injection.

The next difference between the observations of Vera-Lastra et al. [23] and the current work is the fact, that in the discussed study, one of the inclusion criteria was a clinical evidence of autoimmune disease or non-specific autoimmune manifestation altogether with positive autoantibodies reaction. In current work, we incorporated all patients after breast enhancement with hydrogel without confirmation of autoimmune disease and evaluated the incidence of possible ASIA clinical features in this group.

In another study, the authors presented a case of leukopenia after the injection of a large amount of PAAG in the epicranial aponeurosis [24] and concluded that extensive PAAG migration may cause not only local complications but also a long-lasting autoimmune reaction with general complications.

Injection of fluid chemical substances to model the body is a frequent practice in Latin American and Eastern European countries [15, 23], however, in Poland it is a relatively new practice, as mentioned above, in place since 2012. Nevertheless, the results obtained may not be fully reliable because patients with weak and non-specific symptoms are less motivated to seek medical care. Therefore, our group may not reflect the morbidity in the whole polyacrylamide patients population.

Jara et al. [25] have recently published a comprehensive review, in which the articles published from 2011 to 2016 dedicated to the cases of ASIA syndrome were analyzed. The authors found that, so far worldwide, out of 4479 confirmed cases of ASIA, 305 fulfilled the criteria of severe disease and 11 patients died. Among the main causes of severe manifestations, HPV or influenza vaccination, silicone, and mineral oil injections were mentioned. Some authors explained these differences by complex genetic and individual factors that affect the final autoimmune manifestation [26, 27].

Tervaert and Kappel [28] examined a group of women with silicone implant incompatibility syndrome with concomitant symptoms analogous to ASIA. Median time between the implantation of silicone breast implants and the beginning of complaints was 10 years, varying from 2 to 24 years. According to the authors, a long interval between the implant operation and the onset of symptoms could be explained by the fact that silicones need to migrate out of the breasts to induce a substantial adjuvant effect. In our study, the median time between the implantation of PAAG and the onset of complaints was much shorter—about 1.5 years, ranging from 0 to 60 months. This difference can probably be justified by the enormous tendency of hydrogel to migrate and disperse, compared to the possibility of leakage from the implant, so that its particles can be carried easily to local and distal lymph nodes, promoting a generalized autoimmune reaction. According to Brown et al. [29], the possibility of autoimmune diseases increases with breast implant rupture and silicone bleed incidence. These arguments may explain a vast percentage of ASIA criteria fulfillment after the use of fluid breast fillers, observed in our group, compared to the symptoms observed after typical silicone breast implants.

The small size of the study group as well as an observational character of this study constitute limitations of the presented research.

Considering the severity of some of the complications studied, it is worth mentioning, that in January 2020 PAAG—due to the decision of the Office for Registration of Medical Products, Medicinal Devices and Biocidal Products, was disqualified from the use in plastic surgery in Poland [20].

Summing up, in recent years, extensive invasive surgical procedures have subsided in favor of minimally invasive procedures using newer technologies, such as laser and materials, e.g. fillers. It was assumed that this should lead to faster recovery, reduction in pain and risk of complications. However, due to the tendency to reduce costs, these procedures are often performed with unverified injection materials. Even if they are carried out using the approved fillers, complications should be carefully monitored and catalogued. The exchange of observations and experience between surgical centers may indicate optimal options for using a particular material in plastic surgery, as well as provide arguments to stop using it—whenever it becomes necessary. In the case of PAAG, currently widely used material, observations should be broadened to determine its impact on the development of autoimmune phenomena.

The preliminary observations included in the present study require group extension and follow up control studies with symptoms assessment and further immunological tests. The autoimmune reactivity of PAAG requires more thorough research. Nevertheless, we believe that the results of this study may have significant implications for the general welfare of patients undergoing plastic surgery procedures.

## Conclusions

Polyacrylamide hydrogel breast filling carries the risk of developing ASIA symptoms. Its removal may bring improvement in some cases. Therefore, the assessment of general symptoms and screening for autoimmune/autoinflammatory manifestations should be an obligatory part of the examination of every patient after PAAG breast augmentation.

## Data Availability

All data and material support the published claims and comply with field standards.
